# Genome resequencing and custom genotyping elucidates the origin and dissemination history of an emblematic grapevine cultivar, ‘Tempranillo Tinto’

**DOI:** 10.1093/hr/uhaf237

**Published:** 2025-09-03

**Authors:** Javier Tello, Pablo Carbonell-Bejerano, Rafael Torres-Pérez, Yolanda Ferradás, Carolina Royo, Javier Portu, José Félix Cibriáin, Juan Carlos Oliveros, Javier Ibáñez, José Miguel Martínez-Zapater

**Affiliations:** Instituto de Ciencias de la Vid y del Vino (CSIC, Universidad de La Rioja, Gobierno de La Rioja), Finca La Grajera, Ctra. de Burgos Km. 6, 26007 Logroño, Spain; Instituto de Ciencias de la Vid y del Vino (CSIC, Universidad de La Rioja, Gobierno de La Rioja), Finca La Grajera, Ctra. de Burgos Km. 6, 26007 Logroño, Spain; Bioinformática para Genómica y Proteómica, Centro Nacional de Biotecnología (CSIC), C/Darwin 3, 28049 Madrid, Spain; Instituto de Ciencias de la Vid y del Vino (CSIC, Universidad de La Rioja, Gobierno de La Rioja), Finca La Grajera, Ctra. de Burgos Km. 6, 26007 Logroño, Spain; Facultade de Bioloxía, Universidade de Santiago de Compostela, 15872 Santiago de Compostela, Spain; Instituto de Ciencias de la Vid y del Vino (CSIC, Universidad de La Rioja, Gobierno de La Rioja), Finca La Grajera, Ctra. de Burgos Km. 6, 26007 Logroño, Spain; Instituto de Ciencias de la Vid y del Vino (CSIC, Universidad de La Rioja, Gobierno de La Rioja), Finca La Grajera, Ctra. de Burgos Km. 6, 26007 Logroño, Spain; Sección de Viticultura y Enología de Gobierno de Navarra-EVENA, C/Valle de Obra 34, 31390 Olite, Spain; Bioinformática para Genómica y Proteómica, Centro Nacional de Biotecnología (CSIC), C/Darwin 3, 28049 Madrid, Spain; Instituto de Ciencias de la Vid y del Vino (CSIC, Universidad de La Rioja, Gobierno de La Rioja), Finca La Grajera, Ctra. de Burgos Km. 6, 26007 Logroño, Spain; Instituto de Ciencias de la Vid y del Vino (CSIC, Universidad de La Rioja, Gobierno de La Rioja), Finca La Grajera, Ctra. de Burgos Km. 6, 26007 Logroño, Spain

## Abstract

Grapevine cultivars are vegetatively propagated to maintain their varietal characteristics. However, long periods of cultivar multiplication result in the accumulation of spontaneous somatic mutations that can differ among clonal lines. Here, we explored this intravarietal genetic diversity to trace back the origin and dissemination history of ‘Tempranillo Tinto’, the third most cultivated wine grape variety worldwide. A stringent somatic variant calling over whole-genome resequencing data of 35 ‘Tempranillo Tinto’ grapevines from seven Iberian winemaking regions revealed 158 somatic single nucleotide variants (SNVs) shared by some of the plants. Among them, 56 highly informative SNVs were used to custom-design a high-throughput intravarietal genotyping assay, which was validated and used to analyze 185 vines representing a broader geographic distribution. Phylogenetic analyses revealed three major clonal lineages in ‘Tempranillo Tinto’ that grouped the samples according to their geographic origin. By inferring the ancestral SNV alleles in ‘Tempranillo Tinto’ from whole-genome resequencing data of its parents, we determined the Ebro River Valley in Northeast Spain as the most likely birthplace of the cultivar. Derived alleles revealed one major historical human-mediated westward dissemination route from this original site towards the winemaking regions following the Duero River Valley and then, to the South in Portugal. Clonal lineages also revealed the polyphyletic nature of somatic variant traits of interest for grape and wine quality production under climate change conditions. Our findings elucidate the origin and historical dispersal of ‘Tempranillo Tinto’ and underscore genomic strategies for advancing clonal improvement to ensure the sustainability of valuable traditional grapevine cultivars.

## Introduction

Vegetative propagation has been used in grapevine cultivation since ancient times, as already indicated by the Roman author Columella in the 1st century CE [1]. This strategy of multiplication is used to maintain varietal attributes, and it has allowed the preservation of ancient grapevine cultivars for centuries [2] and their spread all around the world [3]. Continuous cultivation and vegetative multiplication of traditional grapevine cultivars gives rise to the progressive accumulation of somatic genome variations, which stack across repetitive cycles of asexual propagation. Somatic variation is used for grapevine improvement, as it provides a relevant source of variability for clonal selection programs, prompting the adaptation of emblematic cultivars to new needs [4], including those related to current climate conditions and novel consumer preferences [5]. Somatic mutations range from single nucleotide variants (SNVs), small insertions and deletions (INDELs) and variable transposon insertions, to large structural rearrangements [6]. In-depth studies have linked some of these somatic mutational events to relevant phenotypic changes, like the SNV originating the seedless phenotype in ‘Sultanina’ grapes [7], or the complex chromosome reshuffling causing loss of berry color and low gamete viability in ‘Tempranillo Blanco’ cultivar [8, 9]. However, somatic mutations are relatively scarce in the coding regions of genes relative to introns and intergenic space [6, 10, 11] and most of them are silent or behave as recessive mutations, so their overall impact on the phenotype of the cultivar is low. In fact, most of the plants obtained by vegetative propagation are phenotypically identical to the plant of origin, and plants showing phenotypic variation due to somatic variation are only occasionally observed [12].

Current estimations indicate that there are between 6000 and 10 000 wine grape cultivars across the globe [3]. However, although global genetic diversity is high, wine grape world production relies on a very limited number of extensively-grown cultivars [13]. One of them is ‘Tempranillo Tinto’, which is Spain’s flagship red wine grape cultivar. Its cultivation in Spain can be reliably traced back to the end of the 18th century CE [14–16], and it is now cultivated in *ca*. 200 000 ha across the country, representing 21.4% of the Spanish vineyard surface (https://www.mapa.gob.es/es/). In addition, it is the most commonly grown cultivar in Portugal, spread in about 18 000 ha, mainly found in the Alentejo and Douro winemaking regions [17]. A proof of the long cultivation of ‘Tempranillo Tinto’ in the Iberian Peninsula is the many synonyms traditionally used for its designation in different regions, which include ‘Tinta del País’, ‘Cencibel’, ‘Tinto Fino de Madrid’, ‘Tinta de Toro’, ‘Arauxa’, and ‘Ull de Llebre’ in Spain, and ‘Aragonez’ and ‘Tinta Roriz’ in Portugal [18]. Genetic analyses revealed ‘Benedicto’ and ‘Albillo Mayor’ as the parents of ‘Tempranillo Tinto’ [18]. ‘Albillo Mayor’ is a white-berried grapevine cultivar found nowadays in few hectares in the north-central area of the Iberian Peninsula [19]. ‘Benedicto’ is a less known black-berried grapevine cultivar with functional female flowers, occasionally found as relict plants in old vineyards of the northern and central areas of the Iberian Peninsula [20, 21]. The narrow distribution of ‘Benedicto’ and its role as mother of ‘Tempranillo Tinto’, pointed out the Ebro River Valley as the region where ‘Tempranillo Tinto’ could have spontaneously originated [18]. This theory agrees with the historical records available for this cultivar [14, 16]. Besides, the lack of reliable written references to ‘Tempranillo Tinto’ before the 18th century indicates that this cultivar could have a relatively recent origin [18]. As for other cultivars [22], ‘Tempranillo Tinto’ was firstly propagated by positive mass selection for yield-related traits. This process drove to high genetic variability and high phenotypic heterogeneity in plants within a single vineyard, still observable nowadays in old vineyards of the Iberian Peninsula [5, 17, 23]. From the 1980s, ‘Tempranillo Tinto’ improvement was driven by clonal selection programs aimed to multiply a limited number of vines with superior features [24]. Even though this process improved the sanitary quality of the plant material available for this emblematic cultivar, this rigorous selection was likely linked to a loss of the clonal genetic diversity initially available in the vineyards [12].

Current genomics-based approaches allow addressing old questions. Whole-genome resequencing (WGR) data and tailored variant calling procedures can efficiently detect somatic variations among grapevines of the same cultivar. This information can be useful to determine their genetic relationships, which arrange in lineages that group samples of high genetic similitude, separated by somatic variations [10, 11, 25]. If combined with historical planting records or with information tracing back the dispersal of grapevines in wide regions, these somatic markers can contribute to understand the historical propagation processes experienced by emblematic grapevine cultivars in important winemaking regions. For example, Calderón *et al.* [10] identified a series of SNVs that pointed out the existence of two major clonal lineages in ‘Malbec’ (syn. ‘Côt’) that associated with the different propagation processes experienced by this cultivar in Europe and in South America. Similarly, Gambino *et al.* [11] discovered a series of somatic markers for ‘Nebbiolo’ that differentiated some major genotypes of different geographic regions, and Roach *et al.* [25] unveiled how the ‘Chardonnay’ clone ‘Gingin’ appeared in Australia.

Today, the viticulture sector faces unprecedented challenges, with climate change compromising grape production, fruit composition and wine quality in many traditional winemaking regions [26]. The use of grapevine varietal diversity can reduce losses from climate change [27], but the use of alternative cultivars is limited by the rigid regulations of wine geographical indications [28]. Despite being one of the most extended grapevine cultivars in the world [13], some of the varietal characteristics of ‘Tempranillo Tinto’ (like its early ripeness and short vegetative cycle) mismatch with current warming conditions in its traditional cultivation regions, compromising its use for high quality winemaking in the near future [29]. Thus, it is of utmost relevance identifying new clones with late ripening and low sugar levels for the improvement of this cultivar [5]. In this line, the combination of phenotypic data with phylogenomic approaches has been useful in vegetatively propagated crops like apple (*Malus domestica*) and sweet orange (*Citrus sinensis*) to determine whether clones with beneficial characteristics derived from a common variant ancestor or arose in independent events [30–32]. This information can be useful to guide current and future strategies for cultivar improvement.

The aim of this study was to explore the intravarietal genomic diversity extant within ‘Tempranillo Tinto’ and its possible relationship with the propagation and selection history of the cultivar. To that aim, we resequenced the whole genome of ‘Tempranillo Tinto’ plants collected from different Iberian winemaking regions and searched for intra-varietal SNVs discriminating clonal lineages. The design of a custom high-throughput assay for SNV genotyping enabled the analysis of a large number of plants of ‘Tempranillo Tinto’. Phylogenetic analyses revealed the most likely region of origin of this emblematic cultivar, and its main dissemination routes throughout the Iberian Peninsula. We also located the clonal lineages of origin of selected somatic variant phenotypes as a strategy to foster clonal improvement and intravarietal diversity conservation.

## Results

### Genomic variation in ‘Tempranillo’ cultivar

**Figure 1:**
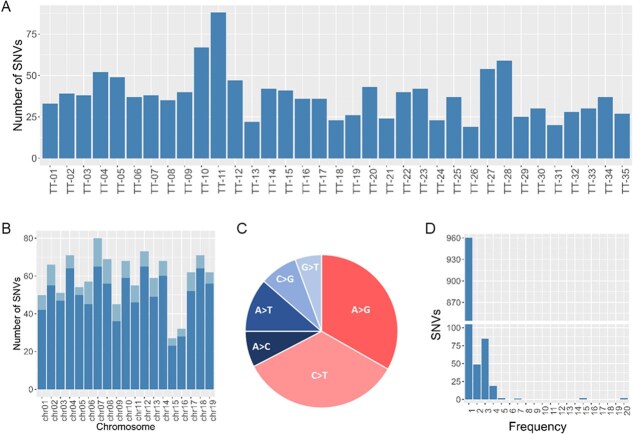
Single nucleotide variants (SNVs) detected among ‘Tempranillo’ grapevines. **(A)** Number of SNVs detected per ‘Tempranillo Tinto’ (TT) grapevine, coded as indicated in Table S1  **(B)** Number of SNVs detected per chromosome. Sample-specific SNVs are indicated in dark blue; shared SNVs are shown in light blue. **(C)** Number of SNVs detected per type of nucleotide variant. The two transition types are shown in different shades of red; the four transversion types are shown in different shades of blue. **(D)** Number of plant-specific (frequency = 1) and shared (frequency > 1) SNVs.

We produced WGR data for 35 ‘Tempranillo Tinto’ grapevines that were selected according to their age and distinct geographical Iberian origin (Table 1, Table S1). Variant calling over WGR data identified 1120 somatic SNVs. The number of called SNVs varied from 19 to 88 per sample (Fig. 1A), and they were well distributed across the 19 chromosomes of the ‘Benedicto’-inherited haplophase assembly of ‘Tempranillo Tinto’ used as reference genome (Fig. 1B, Table S2). Agreeing with the reported signatures of somatic SNVs in other vegetatively propagated crop species [30, 33, 34], the two transition types were more abundant than the four transversion types, 67.4% versus 32.6% (Fig. 1C). C > T were the most frequent SNVs among transition types (383 mutations, 34.2%), and A > T SNVs were the most abundant among the detected transversions (127 mutations, 11.3%). As expected for novel somatic mutations [6, 35], all detected SNVs were heterozygous. SnpEff prediction revealed that 90.7% (1016 SNVs) of the SNVs had no effect, whilst 93 (8.3%) and 11 (less than 1%) SNVs were predicted to have moderate and high effects on the coding sequence, respectively (Table S2). After screening these 1120 genomic positions in 15 additional ‘Tempranillo’ samples from available WGR data, 960 SNVs (85.7%) were found specific to single samples (Fig. 1D). The remaining SNVs (160, 14.3%) were found in multiple samples, most of them in two (49 SNVs, 4.4%), three (85 SNVs, 7.6%) or four (19 SNVs, 1.7%) samples. Considering their higher resolution for phylogenetics, we focused our research in the set of 160 shared somatic SNVs, whose reliability was individually assessed by means of IGV [36] (see Fig. S1 for representative examples). This curation process led to remove two SNVs that did not have data in every sample (B_chr03_20048070 and B_chr06_13650891). As a result, a set of 158 highly-confident somatic SNVs was used for further analyses (Table S2).

**Table 1:** Origin of the 185 Iberian ‘Tempranillo’ grapevines studied in this work

**Wine geographical indication**	**Code**	**Region**	**Samples**	**Samples for WGR** ^**a**^
D.O.Ca. Rioja	RJ	North Spain (Ebro River Valley)	110	15
D.O. Cigales	CG	North West Spain (Duero River Valley)	4	0
D.O. La Mancha	LM	Central Spain	1	1
D.O. Navarra	NV	North Spain (Ebro River Valley)	3	3
D.O. Penedès	PN	North West Spain (Ebro River Valley)	2	0
D.O. Ribera del Duero	RD	North Spain (Duero River Valley)	20	6
D.O. Toro	TO	North Spain (Duero River Valley)	15	3
D.O. Tarragona	TR	North West Spain (Ebro River Valley)	2	0
D.O. Valencia	VL	Eastern Spain	2	0
D.O. Vinos de Madrid	VM	Central Spain	1	0
D.O.P. Cariñena	CR	North East Spain (Ebro River Valley)	2	0
R.D. Douro	DO	North Portugal (Duero River Valley)	4	4
Vinhos do Alentejo	AL	South Portugal	4	3
Unknown	Unk	Uncertain	15	0

a Whole-genome resequencing

### Revealing the identity of the ancestral ‘Tempranillo’ genotype

Having confidently established 158 somatic SNVs, we identified up to 20 different genotypes (GT-00 to GT-19) within the 50 (35 + 15) ‘Tempranillo’ grapevine genome sequences explored in this work (Table S3). The most frequent one (GT-00) was found 11 times. Then, three genotypes (GT-12, GT-13, and GT-16) were found four times, four genotypes three times (GT-02, GT-03, GT-07, and GT-10), and three genotypes twice (GT-08, GT-15, and GT-19). The remaining nine genotypes were identified as singletons (Table S3).

**Figure 2:**
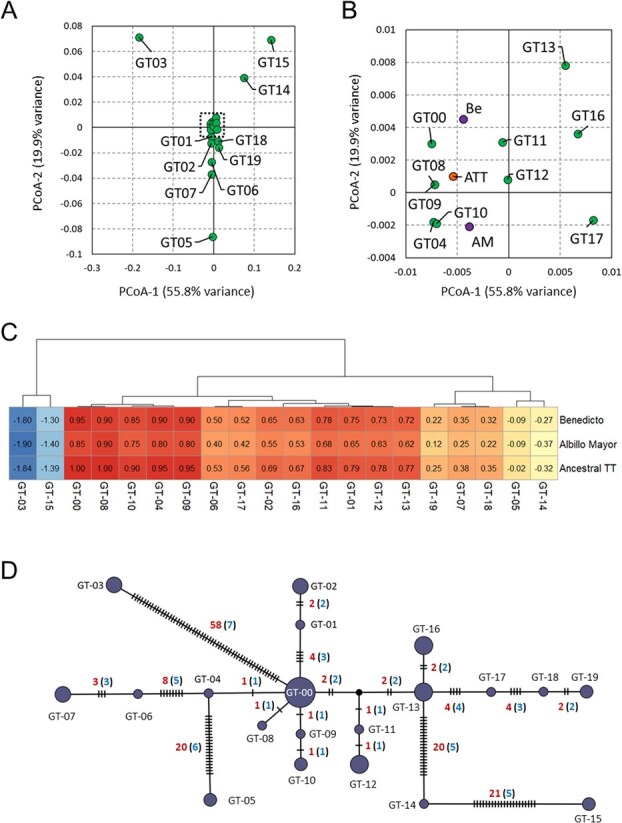
Relationships between 20 ‘Tempranillo’ genotypes (GT), based on 158 SNVs screened in 50 grapevines. **(A)** Principal coordinate analyses from a Euclidean dissimilarity matrix obtained between 20 ‘Tempranillo’ genotypes (in green), their progenitors (‘Albillo Mayor’ and ‘Benedicto’), and the *in silico* ‘Ancestral Tempranillo’ genotype inferred from genetic data of ‘Albillo Mayor’ and ‘Benedicto’. **(B)** Zoomed-in area of the dashed rectangle indicated in **A**. ‘Benedicto’ (Be) and ‘Albillo Mayor’ (AM) are shown in purple; the *in silico* ‘Ancestral Tempranillo’ ATT, in orange. **(C)** Pairwise relatedness values obtained between 20 ‘Tempranillo’ genotypes and ‘Benedicto’, ‘Albillo Mayor’, and an inferred ‘Ancestral Tempranillo’ (Ancestral TT) genotype. Genotypes are clustered according to an Euclidean distance matrix based on kinship coefficients obtained as described in Material and Methods, and coloured following a blue-to-red scale. Values in red indicate a high relationship between two genotypes; values in blue indicate low relationship. **(D)** Phylogenetic network between 20 ‘Tempranillo Tinto’ genotypes, which are shown as dark-colored circles, whose size is proportional to its frequency across the 50 analyzed ‘Tempranillo Tinto’ samples. Small black circles indicate inferred intermediate genotypes not detected in the 50 resequenced samples. The marks crossing each phylogenetic branch indicate the number of SNVs differentiating two contiguous genotypes, indicated in red. The number of SNVs selected from each phylogenetic line to custom-design an intra-varietal genotyping assay for ‘Tempranillo’ are indicated in blue, between brackets.

The 20 ‘Tempranillo’ genotypes were used for a PCoA to unravel patterns of genetic relatedness. This analysis also included an *in silico* ancestral genotype of ‘Tempranillo Tinto’ (ATT), inferred from genetic data of ‘Albillo Mayor’ and ‘Benedicto’ (the parents of ‘Tempranillo Tinto’) at the 158 SNVs, excluding the three positions in which one or both parent cultivars were found to be heterozygous (B_chr02_14844060, B_chr12_16426387, B_chr19_11789116) (Table S3). PCoA identified five genotypes (GT-00, GT-04, GT-08, GT-09, and GT-10) as the closest ones to ATT, as well as to the genotypes of ‘Albillo Mayor’ and ‘Benedicto’ (Fig. 2A and B). In agreement with this global distribution, these five genotypes obtained the highest pairwise kinship coefficients with ATT and both parents of ‘Tempranillo Tinto’ (Fig. 2C). Among them, GT-00 and GT-08 had the highest pairwise kinship coefficients with ATT. Considering the 158 SNVs analyzed, GT-00 and GT-08 differed in only one SNV (B_chr19_11789116), being GT-08 heterozygous (C:G) and GT-00 homozygous (C:C) (Table S3). The other 18 ‘Tempranillo’ genotypes were also homozygous (C:C) at this SNV. Although the original ‘Tempranillo Tinto’ grapevine had the same probability of being homozygous or heterozygous at B_chr19_11789116 (‘Albillo Mayor’ is heterozygous, C:G, and ‘Benedicto’ homozygous, C:C), the uniqueness of the C:G genotype at this SNV position in GT-08 (and the appearance of novel somatic variations most likely as heterozygous mutations [6, 35]) suggests that GT-08 derived from GT-00. Therefore, GT-00 might be considered as the putative ancestral genotype of ‘Tempranillo’, a role also supported by its overall high frequency.

The median-joining phylogenetic network inferred for the 20 identified genotypes also identified GT-00 as the putative ancestral genotype of ‘Tempranillo Tinto’. As observed in Fig. 2D, GT-00 positioned at the center of the network, with multiple genotypes directly linked to this major genotype by a different number of SNVs, from one (GT-04, GT-08, and GT-09) to 58 (GT-03). Among all the lineages branching from GT-00, two seem to be more relevant, given the number of genotypes that derived from them. One phylogenetic lineage leads to GT-04 (on the left of the figure), and another to an inferred intermediate genotype not detected in this study (on the right). This distribution suggests that these two firstly derived genotypes acted as relevant points of divergence in the evolutionary diversification of ‘Tempranillo Tinto’.

### Tracing the propagation history of ‘Tempranillo Tinto’ through custom genotyping of key somatic SNVs

**Figure 3:**
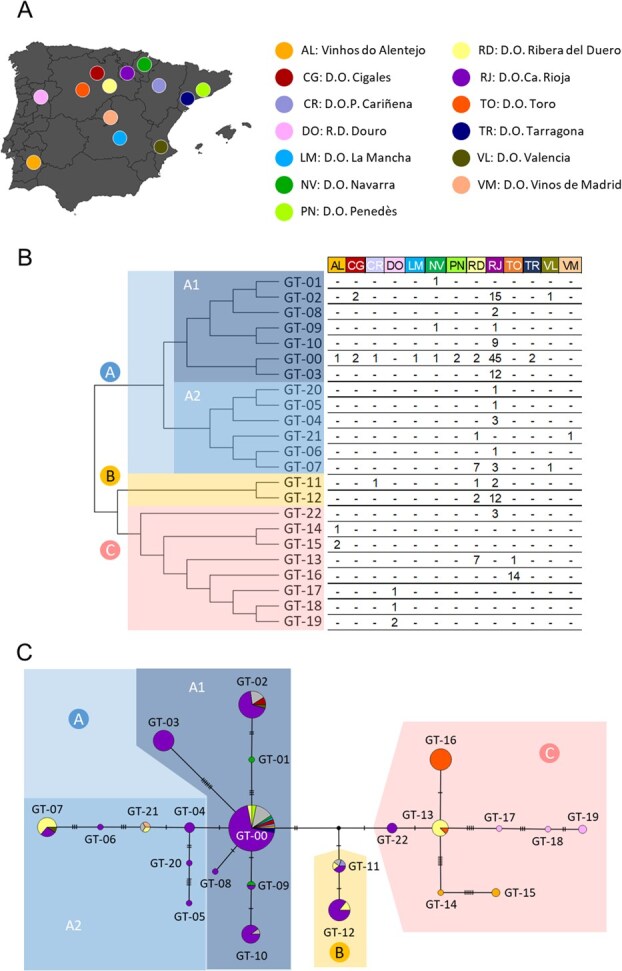
Phylogenetic relationships between 23 ‘Tempranillo’ genotypes, based on 56 SNVs genotyped in 185 grapevines. **(A)** Location of the Spanish and Portuguese wine geographical indications explored in this work. **(B)** Phylogenetic tree based on the neighbor-joining method. The major clades (A, B, C) and subclades (A1, A2) are shown in different colors. The number of grapevines belonging to each genotype is shown for the geographical indications explored (color-coded as indicated in **A**). **(C)** Phylogenetic network, where each circle represents one genotype, whose size is proportional to the number of grapevines belonging to each genotype. Different circle sectors denote the genotype frequency found in different wine geographical indications, as indicated in **A**. Grapevines of uncertain origin are represented in grey. The number of marks crossing each phylogenetic branch shows the number of SNVs differencing two adjacent genotypes. Small black circles indicate inferred intermediate genotypes not detected in this study. The major clades (A, B, C) and subclades (A1, A2) found in **B** are shown in different colors.

As observed in Fig. 2D, the number of SNVs distinguishing two adjacent genotypes varied widely, from just one (e.g. GT-00 → GT-04, GT-00 → GT-08, GT-00 → GT-09, and GT-09 → GT-10) to 20 or more (GT-00 → GT-03, GT-04 → GT-05, GT-13 → GT-14, and GT-14 → GT-15). To prevent the genotyping of less informative SNVs that might have arisen within the same evolution lineage, we reduced the set of detected SNVs to a subset of 56 key somatic SNVs (Table S2), which represented all phylogenetic branches observed (Fig. 2D). These 56 SNVs were used to design a custom high-throughput genotyping assay for ‘Tempranillo’, which was successfully validated in the 50 ‘Tempranillo’ grapevines used for SNV selection (Table S4). This 100% validation rate supports the reliability of both approaches, the variant calling pipeline and the SNV genotyping assay. Thereby, 135 additional ‘Tempranillo’ accessions from 13 Spanish and Portuguese wine geographical indications were genotyped for these SNV markers with our custom assay (Fig. 3A, Table 1 and Table S1). Global data of the 185 genotyped Tempranillo accessions identified 23 different genotypes, including the 20 genotypes previously found plus three new ones, named GT-20, GT-21, and GT-22 (Fig. 3B and Table S5). Phylogenetic analysis grouped all 23 genotypes into three major clades of different size (Fig. 3B). Clade A included 13 genotypes, which distributed in a star-like pattern from GT-00, the putative ancestral genotype of ‘Tempranillo’ (Fig. 3C). This clade could be divided into two subclades (A1 and A2) with a similar size (Fig. 3B). Subclade A1 grouped seven genotypes, including GT-00 and six genotypes derived from GT-00 by a low number of mutations. Subclade A2 grouped six genotypes arranged into two branches deriving from GT-04 (Fig. 3C). GT-04 derived itself from GT-00 by one SNV. Genotypes of clade A were abundantly found in grapevines from the Spanish winemaking regions of D.O.Ca. Rioja, D.O. Navarra, D.O.P. Cariñena, D.O. Penedès, and D.O. Tarragona (all of them in Northern Spain, along the Ebro River Valley), as well as in other regions in Northern Spain like D.O. Ribera del Duero and D.O. Cigales. Clade B contained only two genotypes that were separated from GT-00 by three to four SNVs (Fig. 3C). These two genotypes were mostly found in the Spanish winemaking region of D.O.Ca. Rioja (Fig. 3B). Lastly, clade C grouped eight genotypes (Fig. 3B) that arranged in three evolutionary branches deriving from GT-13 (Fig. 3C). One of these branches included only one genotype (GT-16), which was exclusively found in 14 grapevines of the Spanish winemaking region of D.O. Toro (Fig. 3C). The other two branches grouped three and two genotypes found in the Portuguese winemaking regions of R.D. Douro and Vinhos do Alentejo, respectively (Fig. 3C). Interestingly, GT-13 was mostly found in grapevines from the Spanish winemaking region of D.O. Ribera del Duero (Fig. 3B). GT-13 derived from GT-22 (Fig. 3C), which was exclusively found in grapevines from the Spanish winemaking region of D.O.Ca. Rioja (Fig. 3B).

### Exploring the origin of clonal variation for adaptive and innovative traits in ‘Tempranillo Tinto’

**Figure 4:**
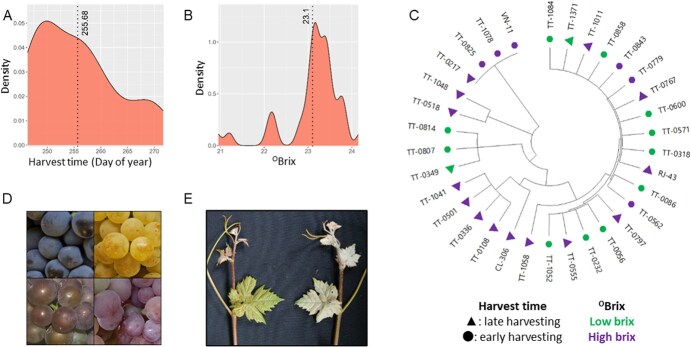
Phenotypic differences between some of the ‘Tempranillo Tinto’ grapevines included in this work. **(A)** Range of phenotypic diversity for the date of harvest observed in 30 clonal selections and three commercial clones of ‘Tempranillo Tinto’. **(B)** Range of phenotypic diversity for sugar content (°Brix) observed at harvest time in 30 clonal selections and three commercial clones of ‘Tempranillo Tinto’. **(C)** Phylogenetic relationships based on 56 SNVs between 30 clonal selections and three commercial clones of ‘Tempranillo Tinto’. Late/early harvesting individuals, and individuals with low/high sugar content at harvest time are graphically represented according to the inset. **(D)** Phenotypic diversity in ‘Tempranillo’ berry-colour somatic variants. From left to right and up to down: original black-berried ‘Tempranillo Tinto’ (sample: Tempranillo-063), white-berried ‘Tempranillo Blanco’ (Tempranillo-174), and two grey-berried ‘Tempranillo Royo’ (Tempranillo-134 and Tempranillo-040) variants. **(E)** Phenotypic differences in the density of prostrate hairs in the original ‘Tempranillo Tinto’ (sample: Tempranillo-063) and the ‘hairy’ ‘Tempranillo Peludo’ (Tempranillo-093).

Harvest date and the content of sugars in ripe grapes are two crucial traits in wine grape production and thus are essential in clonal selection programs. The evaluation of the harvest date in 30 clonal selections and three commercial clones of ‘Tempranillo Tinto’ with wide phenotypic diversity revealed a difference of 25 days between the earliest and the latest clone (Fig. 4A and Table S1). Grape sugar levels in ripe berries varied widely too, from 20.9 to 24.2°Brix between the clone with lowest and highest sugar content at harvest time (Fig. 4B and Table S1). Their phylogenetic relationship (based on 56 key somatic SNVs) placed some candidates with favorable traits for quality wine production in warming climate (like late harvest time or low grape sugar levels) in different clades, suggesting that they originated from multiple independent mutational events (Fig. 4C). In other cases, clone candidates with favorable features were predominant in certain clades, suggesting that they may derive from a common ancestor with already improved characteristics. For example, clone candidates TT-0349, TT-0807, and TT-0814 (all with genotype GT-02) had lower than average grape sugar levels at harvest time. Similarly, clone candidates TT-0581 and TT-1048 (with genotypes GT-06 and GT-07, respectively, separated by three SNVs (Fig. 3C)), and clone candidates TT-0108, TT-0336, TT-0501, and TT-1041 (all with genotype GT-12) were harvested later than the average. Lastly, we identified that the ten accessions of ‘Tempranillo Royo’ (somatic variant with grey-skinned berries, Fig. 4D) have two different genotypes (GT-00 and GT-10), suggesting at least two independent origins for their reduced anthocyanin pigmentation phenotype (Table S1). The in-depth analysis of 48 SNPs in these grapevines to detect the extent of the loss of heterozygosity (LoH) produced by deletions in the berry color locus in chromosome 2 revealed five different hemizygous deletion patterns (Fig. S2). Interestingly, the LoH patterns found in the grapevines with GT-00 (patterns I, II, II, and IV) and in the one with GT-10 (pattern V) were found to be different, proving the independent origin of their reduced anthocyanin pigmentation phenotype, which supports phylogenetic results. In contrast, the three accessions of ‘Tempranillo Peludo’ (somatic variant with a ‘hairy’ leaf phenotype, Fig. 4E) analyzed in this work have the same genotype, GT-02 (Table S1).

## Discussion

Somatic mutations accumulate in crop species where varieties are cultivated for long time and are multiplied by vegetative propagation, like the grapevine [12, 30, 32, 33]. This process has generated multiple and diverse somatic variants in grapevine cultivars with long history and/or large extension of cultivation [37]. Some of these variants can have new phenotypes of interest for cultivar improvement and adaptation to current conditions, and they are the basis for the development of new commercial clones. Regardless their phenotypic impact, the study of the intra-varietal genetic diversity can aid to reveal the complex history of grapevine cultivars of cultural relevance in their traditional cultivation areas [10, 11, 25]. Previous studies aimed at quantifying the extant of SNV intra-varietal grapevine diversity indicated wide varietal differences, ranging from the detection of few to thousands of SNVs [6, 10, 11, 22, 25, 38]. The detected SNV diversity depends on intrinsic factors like the number and genetic similarity of the grapevines used for SNV detection, or the age and historical cultivation area of the variety [6, 10, 11]. Technical considerations like the DNA sequencing technology and the bioinformatics approach for SNV calling also influence the number and quality of variants identified [39, 40]. Here, we resequenced the genome of 35 old ‘Tempranillo Tinto’ grapevines from distinct Iberian winemaking regions where it has been cultivated for centuries, to maximize the detection of intra-varietal diversity within this cultivar. In addition, the use of the ‘Benedicto’ haploid complement of the genome assembly of ‘Tempranillo Tinto’ provided an accurate reference for the alignment of WGR reads of the ‘Tempranillo Tinto’ samples used for high quality SNV discovery. These factors, combined with a stringent variant calling algorithm and subsequent filters used to minimize false positive findings, successfully allowed obtaining enough highly-confident SNVs for the objective of this work. The 100% validation of the somatic SNVs selected for our custom genotyping assay support the high accuracy of the somatic SNV calls we obtained. However, we assume that the false negative rate in our approach should be large considering that more than half of the genome assembly, involving repetitive sequence, was excluded by our calling filters. Interestingly, there were limited shared somatic SNVs between ‘Tempranillo Tinto’ samples (Fig. 2C). As previously reported after the analysis of 15 ‘Chardonnay’ clones [22], this finding likely derives from the long propagation history of ‘Tempranillo Tinto’ by mass selection, which resulted in a high level of intra-varietal genetic diversity. This process translates in a clear genetic distinction between the many derived genotypes of ‘Tempranillo Tinto’ that originated as isolates from ancient vineyards. Besides, a reduced set of shared somatic SNVs was used to develop an effective and cost-efficient genotyping system for the intra-varietal differentiation of ‘Tempranillo’ grapevines. This approach aided us to determine the likely region of origin and the main dispersal routes of this cultivar in the Iberian Peninsula. We could also explore whether traits of interest to adapt this cultivar to current climate conditions have a monophyletic or polyphyletic origin, what can be of interest to guide clonal selection programs.

### ‘Tempranillo Tinto’ likely originated in the upper or the middle course of the Ebro River Valley

Previous studies based on the analysis of SSR, retrotransposons, or AFLP markers indicated that today’s genetic diversity of ‘Tempranillo Tinto’ is the result of an intense vegetative propagation and further distribution of a reduced number of genotypes [41, 42]. Agreeing with these results, here we found that most ‘Tempranillo’ intra-varietal genotypes based on shared somatic SNVs arranged in a phylogenetic network that followed a star-like topology (Fig. 3C), which in sexually reproducing species is indicative of a population of recent origin in exponential growth [43]. Here, this pattern might be reflecting the intensive vegetative multiplication that ‘Tempranillo Tinto’ experienced during its initial mass selection, which was necessary to expand the cultivar into new winemaking regions. Indeed, given that most of the grapevines from our sampling used for WGR can be traced back to a time before clonal selection programs for ‘Tempranillo Tinto’ started [44], most of the genotypes identified in this study might correspond to those deriving from the early propagation efforts done by positive mass selection. During this initial stage, grapevines with a good production behavior were likely chosen by farmers to expand ‘Tempranillo Tinto’ grape production to other regions. Continuous rounds of vegetative multiplication in different regions would have generated additional somatic variation, contributing in some cases to improve adaptation to the local growing and climate conditions [45]. Then, these derived genotypes might have been subsequently multiplied in a repetitive sequential process that generated the overall genetic diversity available today.

**Figure 5:**
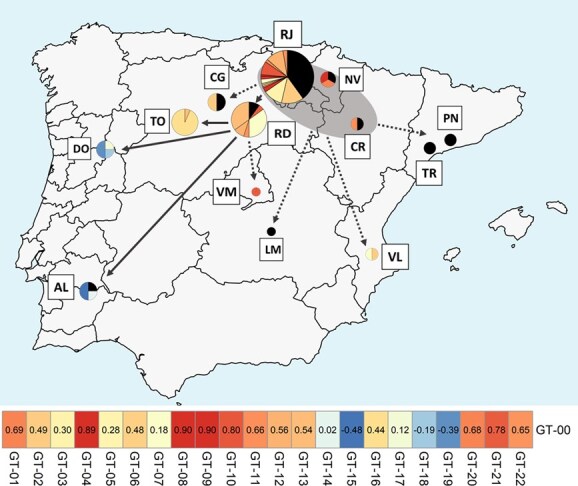
Geographic distribution of 23 ‘Tempranillo’ genotypes across the Iberian Peninsula. Pie charts represent the relative proportions of 23 different genotypes (identified from 56 single-nucleotide variants (SNVs)) in 170 plants sampled from 13 Spanish and Portuguese wine geographical indications (coded as in Table 1). The size of each pie chart is proportional to the number of plants analyzed in each region. The putative ancestral genotype (GT-00) is shown in black. The remaining 22 genotypes are color-coded based on their genetic relatedness to GT-00, as detailed in the inset. Genotypes closely related to GT-00 are displayed in shades of red, while more distant genotypes are shown in shades of blue. The hypothesized region of origin for ‘Tempranillo’ is indicated with a grey shading. Arrows depict the proposed dissemination routes: solid lines for the primary route and dotted lines for suggested secondary routes.

Results in other plant species indicated a positive link between the frequency and antiquity of the clonal genotypes found in a specific region, with the most abundant genotype being the ancestral one [46, 47]. This connection has proven valuable for the indirect identification of the ancestral genotype of the ‘Nebbiolo’ cultivar, which was found to be widely distributed throughout the Italian Piedmont winemaking region [11]. Here, the information retrieved from genome sequencing data from ‘Benedicto’ and ‘Albillo Mayor’ (the two parents of ‘Tempranillo Tinto’) evidenced that GT-00 is the most ancestral genotype of this cultivar, and it was also the most frequent in our study. This role was also in line with the results obtained by PCoA, pairwise kinship coefficients, and phylogenetic analyses. Altogether, our findings indicate the ancestral role of GT-00, from which the remaining 22 genotypes identified in this study derived through successive somatic mutation. Local agrarian records from the 17th century already mention the cultivation of a black-berried grapevine cultivar known as ‘Tempranillo’ in vineyards along the upper and medium course of the Ebro River Valley [48]. While it remains unclear whether this mentions refer to the modern ‘Tempranillo Tinto’, later cultivar descriptions by Valcarcel [16], de Asso [15], and Clemente y Rubio [14] support that it was being widely grown in vineyards of the upper and middle course of the Ebro River Valley by the late 18th century. Today, both ‘Benedicto’ and ‘Albillo Mayor’ are still present as relict plants in this area [20, 21], suggesting some previous relevance in the region that might had enabled the open pollination that gave rise to the cultivar ‘Tempranillo Tinto’ [18]. Supporting these notions, the ancestral genotype GT-00 was found across the five Spanish winemaking regions located along the Ebro River Valley (Fig. 5). Although this ubiquity hinders narrowing down the area where ‘Tempranillo Tinto’ originated, it is expected to find additional highly related genotypes together with the ancestral genotype in the birthplace of a cultivar [11], as they will be the result of the first mass selection processes done to preserve its varietal features [49]. In this line, GT-00 was found with other ancient genotypes in the winemaking regions located at the upper and middle course of the Ebro River Valley (D.O.Ca. Rioja, D.O. Navarra, and D.O.P. Cariñena) (Fig. 5). Indeed, all the genotypes deriving directly from GT-00 (GT-01, GT-03, GT-04, GT-08, and GT-09) were exclusively found in these three winemaking regions (Fig. 3B). These evidences indicated that the ancestral genotype of ‘Tempranillo Tinto’ could have been cultivated for a long time in the upper and middle course of the Ebro River Valley, pointing this area as the most likely region of origin of this traditional cultivar (Fig. 5).

On the contrary, the genotype GT-00 was the only one detected in the two regions located at the lower course of the Ebro River Valley (D.O. Penedès and D.O. Tarragona, Fig. 5). Although this result might be biased by the low number of grapevines screened in these two regions or by the fact that they were not used for SNV discovery (Table 1), this finding likely reflects a more recent introduction of the ancestral genotype of ‘Tempranillo Tinto’ into this area, with a subsequent more limited temporal capability for somatic mutation, diversification and establishment. Indeed, some of the earliest reports recording the presence of ‘Tempranillo Tinto’ in this region indicated that this cultivar was introduced in vineyards of the D.O. Penedès at the end of the 19th century to overcome the grape phylloxera crisis, using cuttings from plants of the D.O.Ca. Rioja winemaking region [50]. GT-00 was also found in other winemaking regions, like the D.O. Cigales and the D.O. Ribera del Duero, in the Spanish northern central plateau (Fig. 5). However, phylogenetic analyses revealed that the other genotypes found in these two regions derived from other intermediate genotypes, which derived themselves from genotypes only found in the region of origin of ‘Tempranillo Tinto’ (Fig. 3). Therefore, the presence of GT-00 in these regions might be the result of introductions of ‘Tempranillo Tinto’ plants sourced in its original site. In fact, the earliest reliable historical records of ‘Tempranillo Tinto’ cultivation in this region date back to the 19th century [51]. Finally, the eventual finding of GT-00 in Central Spain (D.O. La Mancha) and Southern Portugal (Vinhos do Alentejo) likely resulted from the direct introductions of ‘Tempranillo Tinto’ vines harboring the GT-00 genotype. This hypothesis is consistent with historical records that document the movement of cuttings from Logroño (now D.O.Ca. Rioja) to southern winemaking regions by the end of the 18th century [52].

### ‘Tempranillo Tinto’ spread across the Iberian Peninsula through a sequential dissemination process

The use of a reduced number of old grapevines, the uncertainty of their origin, technical difficulties, or different cultivar propagation history complexities might have hindered revealing how the historical dissemination of other emblematic grapevine cultivars took place in their areas of relevance [6, 11]. Nevertheless, our approach successfully revealed the main historical dissemination route of ‘Tempranillo Tinto’ across the Iberian Peninsula. The identification of GT-00 and other firstly-derived genotypes across the Iberian Peninsula supports the assumption that the cultivation of traditional varieties in different winemaking regions was prompted through the human-mediated movement of cuttings obtained from mass-selected grapevines [53]. In the case of ‘Tempranillo Tinto’, we found that this process took place as one major sequential route that progressed westwards from its place of origin (the upper and middle course of the Ebro River Valley) towards the main winemaking regions found across the Duero River Valley in Spain and Portugal, and to the South in Portugal (Fig. 5). Interestingly, the pattern of accumulation of somatic SNVs unveiled that this main route started with cuttings from grapevines harboring the genotype GT-22 (Fig. 3C), which in our sampling was exclusively found in vines of old vineyards of the D.O.Ca. Rioja (Table S1). Thus, it is plausible that plant cuttings from this specific region were first transferred to the nearby winemaking region of D.O. Ribera del Duero, which acted as an important centre of distribution of plants to other winemaking regions across the Duero River Valley (D.O. Toro in Spain, and R.D. Douro in Portugal) and to the south in Portugal (Vinhos do Alentejo). During this process, specific regional genotypes arose and were established (like GT-16 in D.O. Toro, GT-14 and GT-15 in Vinhos do Alentejo, and GT-17, GT-18, and GT-19 in R.D. Douro), likely because they were selected for their adaptation to local environmental conditions. Indeed, significant phenotypic differences in traits like must acidity or sugar levels have been reported between ‘Tempranillo Tinto’ commercial clones derived from these different regions [23, 54]. Resemblant clonal lineages suggesting this main historic dissemination route of ‘Tempranillo Tinto’ (Fig. 5) were identified in a variant calling using only an even number of three grapevines per region (Fig. S3), supporting the reliability of this finding.

Beyond this main historical route, our results were useful to detect additional secondary dissemination processes of more recent origin. At the beginning of the 20th century, ‘Tempranillo Tinto’ was spread all over the Iberian Peninsula [55]. Indeed, multiple new ‘Tempranillo Tinto’ vineyards arose at that time to replace the many old vineyards that were affected by the devastating effect of the grape phylloxera, which appeared in Spain in 1878 and caused an irreversible loss of grapevine genetic resources at both cultivar and intra-cultivar levels [56]. Although the general recommendation at that time was to replace these devastated vineyards with cuttings of plants from local vineyards [55], some exchange of ‘Tempranillo Tinto’ grafted cuttings between regions occurred [57]. In this line, our results supported the reported human-mediated transference of cuttings from the D.O.Ca. Rioja to the D.O. Penedès discussed above [50], and were of interest to outline other movements. For example, the finding of the genotype GT-21 in vines of the winemaking regions of D.O. Ribera del Duero and D.O. Vinos de Madrid (Fig. 3B) suggests a likely movement of plant material from the D.O. Ribera del Duero to the D.O. Vinos de Madrid (Fig. 5). Similarly, the two genotypes found in the D.O. Valencia were also found in the D.O.Ca. Rioja (Fig. 3B). Although the presence of these two genotypes in the D.O. Valencia could have derived from the independent introduction of cuttings from different regions, it is likely that they derived from those of the D.O.Ca. Rioja (Fig. 5).

### Traits of innovation and of adaptation of ‘Tempranillo Tinto’ to climate change conditions are present in clonal lines of different evolutionary origin

Our analyses are not aimed to reveal the genetic basis of traits of interest for ‘Tempranillo Tinto’ innovation or adaptation to climate change conditions. This aim will need of deeper analyses combining complementary strategies at the phenotypic, genomic, and transcriptomic levels, among others [8]. However, we could get enough information to reveal the polyphyletic nature of harvest date and fruit sugar concentration levels at harvest time (Fig. 4C), two traits that are key to promote the adaptation of this cultivar to current growing conditions. A similar finding was found for the grey-berried clonal lines of ‘Tempranillo Royo’ analyzed in this study (Table S1). From a practical perspective, the availability of alternative sources of diversity for improving relevant traits is paramount, as it might allow the identification of the best source to use for cultivar improvement and innovation. In fact, the lack of diversity hinders the improvement of trade-off features that could appear linked to a novel somatic variant with a phenotypic trait of interest, as observed for the limited reproductive performance derived from the chromosome rearrangement causing the variant white berry color in the unique clone available of ‘Tempranillo Blanco’ [8, 9].

The two fruit ripening traits explored here are known to be under a highly complex genetic control [58, 59]. Therefore, it is likely that the beneficial features observed in the clonal variants of different phyletic origin arose from mutational events affecting different molecular steps in the same or different pathways with a convergent phenotypic effect. Thus, their comparative study could be informative to reveal alternative genetic and molecular mechanisms involved in the determination of ripening time and sugar accumulation in grapevine. Likewise, the in-depth study of the different ‘Tempranillo Royo’ grey-berried variants can be of interest to identify different mechanisms involved in berry color determination. The major berry color locus of *Vitis vinifera* grapes corresponds to a cluster of *MYBA* transcription factor genes [60] located in chromosome 2 [61]. Grey-berried somatic variants eventually appear from black-berried grapevine cultivars heterozygous for the berry color locus (like ‘Tempranillo Tinto’ [8]), due to deletions of the functional allele of the *MYBA* genes in the L2 meristem cell layer. This causes that the null allele remains in hemizygosity and, consequently, anthocyanin pigmentation occurs only in the L1-derived epidermis and is absent in all other berry skin cell layers derived from the L2 [62–64]. As expected, the comparison of the genotyping results obtained from DNA extracted from leaves (derived from L1 and L2) and from adventitious roots (from L2) indicated that all the grey-berried grapevines included in this work are periclinal chimeras (data not shown). Interestingly, the study of the LoH on chromosome 2 led to the differentiation of five different hemizygous deletion patterns (Fig. S2). Interestingly, the genetic patterns observed in the grapevines with GT-00 and in that with GT-10 were different (Fig. S2). This result proves the independent origin of the reduced anthocyanin pigmentation phenotype observed in the grapevines with GT-00 and GT-10, and indicates that the grey berry color observed in the grapevine with GT-10 is caused by an alteration other than hemizygous deletions removing the functional alleles of the *MYBA* genes in the berry color locus at chromosome 2.

Somatic mutations have played a pivotal role shaping genetic diversity to foster cultivar resilience against biotic and abiotic stresses [45], and they have been selected for their interest towards cultivar innovation [4]. This process has been historically driven by grape growers, by selecting for multiplication those individuals showing a better growth and production behavior, as well as those providing new phenotypic traits. As our results indicate, similar phenotypes have been selected across different clonal lines to establish vineyards with enhanced adaptive or novel potential. This diversity might have ultimately contributed to the development of the currently adapted commercial clones in recent decades. Knowledge on the molecular basis of this variation could orient future approaches to directly improve specific adaptive and innovative traits.

## Conclusion

Traditional grapevine varieties store information on their history within their genomes. Advances in genomics can be used now to reveal valuable insights into the processes that shaped cultivar development at different scales. The description of the intra-varietal diversity of ‘Tempranillo Tinto’ confirmed that it derives from a single ancestral genotype that is now the most frequently found in the upper and middle course of the Ebro River Valley. This finding supports historical records that indicated this region as the birthplace of this iconic cultivar. Moreover, the genotypic screening of a wide number of ‘Tempranillo’ plants from broad geographic origin elucidated part of the historical dissemination process of this cultivar in the Iberian Peninsula, which was eased by the human-mediated and sequential dispersal of different derived genotypes to different regions. Given that mass propagation in different regions might have selected variants adapted to local environments, intra-varietal genetic information can guide clonal selection programs aimed at adapting traditional grapevine varieties to warmer conditions. Interestingly, we found that clones of different phyletic origin hold traits that can be useful to overcome some varietal limiting features of ‘Tempranillo Tinto’ that compromise its cultivation under warmer climate. This genetic diversity should be used as the basis of cultivar improvement. Given the strong link between viticulture and human civilizations, unveiling the information stored in grapevine genomes can be relevant for understanding the history of viticulture adaptation in regions with long winemaking tradition.

## Materials and methods

### Plant material

One hundred and eighty-five ‘Tempranillo’ plants were used for this study, including 171 grapevines of the original black-berried ‘Tempranillo Tinto’ cultivar, one grapevine of its white-berried somatic variant (‘Tempranillo Blanco’), ten grapevines of the grey-berried somatic variant (‘Tempranillo Royo’ or ‘Tempranillo Gris’), and three grapevines of the ‘hairy’ somatic variant (‘Tempranillo Peludo’, with a high density of trichomes on the abaxial side of the leaf blade) (Fig. 4, Table S1). This set included 137 grapevine samples involving 117 different certified commercial clones or current clonal selections. For some commercial clones, several grapevines were analyzed, provided from different nurseries. We also included 45 grapevine samples prospected from old vineyards, and three accessions stored in grapevine repositories (Table S1). For each plant, young leaves were collected and stored at −80°C prior to total genomic DNA (gDNA) extraction.

The date of harvest and the grape total soluble solid content at harvest time (evaluated as °Brix) were annotated for a subset of 30 clonal selections and three commercial clones (Table S1). These evaluations were done during three consecutive seasons (2020, 2021, and 2022), as previously detailed [5], in plants (10 per accession) maintained in a dedicated experimental plot under even conditions, in terms of grafting, pruning, irrigation, and other cultural practices. Seasonal data were modelled by linear regression to obtain the Best Linear Unbiased Prediction (BLUP) values per genotype and trait [65]. BLUP values are reported in the Table S1. Then, clones were considered early or late harvesting clones if their BLUP values were lower or higher than the mean value of the subset, respectively. The same consideration was used to categorize each clone as a low or high brix clone.

### gDNA extraction, varietal identification, and whole-genome resequencing

Leaf samples were grounded into powder in individual mortars using liquid nitrogen. Then, gDNA was isolated from 100 mg of ground leaf powder using the NZY Plant gDNA isolation kit (NZYTech, Lisbon, Portugal), as indicated by the manufacturer. Quantity and purity of gDNA were assessed on a NanoDrop spectrophotometer (Thermo Scientific, Wilmington, DE, USA) and its integrity by electrophoresis. All plants were confirmed to be ‘Tempranillo’ using 13 microsatellite markers (*VVS2*, *VVMD5*, *VVMD7*, *VVMD25*, *VVMD27*, *VVMD28*, *VVMD32*, *VrZAG62*, *VrZAG79*, *VrZAG112*, *VrZAG29*, *VrZAG67*, and *VrZAG83*) [66–69]. Multiplex PCRs, capillary electrophoresis, and fragments size rating were performed as detailed elsewhere [70]. For Illumina WGR, gDNAs were submitted to the Genomics Unit of the Centre for Genomic Regulation (CRG) core facilities. Sequencing libraries were prepared using the Illumina DNA prep kit (Illumina, San Diego, CA, USA), and the resulting sequencing libraries were evaluated for quantity and quality in a Bioanalyzer 2100 (Agilent, Santa Clara, CA, USA). Libraries were then sequenced on an Illumina HiSeq2500 sequencer (Illumina, San Diego, CA, USA), in 125-bp paired-end reads. Illumina adapters and low-quality read ends were removed by means of cutadapt [71] to ensure correct reads alignment in subsequent steps. FastQC v. 0.11.5 [72] was used for an initial evaluation of reads quality.

### Reads alignment, variant calling, and genotyping using independent resequencing data

WGR reads were aligned to the reference ‘Benedicto’ haplophase genome assembly of ‘Tempranillo Tinto’ [73] using the bwa-mem algorithm implemented in BWA v. 0.7.17 [74], using default parameters. Then, SAMtools v. 1.11 [75] was used to compress the alignment files to generate one full genome .bam file per ‘Tempranillo’ sample. Bam files were deposited in the National Center for Biotechnology Information (NCBI) Sequence Read Archive (SRA) database with accession PRJNA1224223. Alignments’ quality was assessed with Qualimap v. 2.2.1 [76], SAMtools v. 1.11 [75], BCFtools v.1.11 [77], and GATK v. 4.0.3 [78]. Then, the BamTools *split* tool [79] was used to split each full-genome .bam file into 19 .bam files, one per scaffold (chromosome) present in the genome used as reference. Freebayes v.1.3.4 [80] was used for variant calling using the ‘Benedicto’ haplophase genome assembly as reference, with the following parameters -p 2, -C 4, -F 0.25, -min-coverage 530, and -g 5300. This approach generated 19 .vcf files per sample (one per scaffold), which were subsequently merged into one single .vcf file per sample, using the *concat* tool implemented in BCFtools v.1.11 [77]. The detected SNVs were then subjected to a highly stringent filtering process that considers a series of criteria that included genotype quality, sequencing depth, strand bias, allele balance, low mappability, and homopolymer runs in the reference for reducing the probability of detecting false positive SNVs, as detailed in the script available at https://github.com/ratope/VariantFilteringCriteriaVvTempranillo. Putative germline polymorphisms were also filtered out as indicated in the script by comparison to short-read sequencing data of the ‘Tempranillo Tinto’ RJ51 clone grapevine used to build the reference genome assembly and to the alternate ‘Albillo Mayor’-inherited haplophase of the ‘Tempranillo Tinto’ genome assembly [73]. This process generated a set of 1120 SNVs (Table S2), which was obtained using bcftools v.1.11 [77]. These SNVs were genotyped in available WGR data from additional ‘Tempranillo’ samples as well as from ‘Albillo Mayor’ and ‘Benedicto’ [8, 73] (Table S1). To this aim, the WGR data of each sample was aligned to the ‘Benedicto’ haplophase of the ‘Tempranillo Tinto’ genome assembly using the *mem* utility of BWA version 0.7.17 [81] with the option -M. The genotype of the target SNV positions was retrieved from the resulting BAM files using the mpileup utility of BCFtools version 1.8 [82] with the options --min-BQ 20 --min-MQ 20 --excl-flags UNMAP, SECONDARY, QCFAIL, DUP. The output genotypes were saved to a .vcf file using the call utility of BCFtools. SnpEff v.4.0 [83] was used to predict the effect of the 1120 detected SNVs.

### Relationship between ‘Tempranillo’ genotypes

The dataset of 1120 SNVs was furtherly sieved to a set of 158 SNVs, keeping those variants successfully screened in the 50 samples and excluding those only found as singletons (Table S2). The 50 biallelic ‘Tempranillo’ genotypes based on 158 SNVs were screened to identify the number of different ‘Tempranillo’ genotypes (GTs) and their frequencies. The overall similarity between those genotypes was evaluated by a Principal Coordinate Analysis in DARwin 6.0 [84] from a dissimilarity matrix based upon Euclidean distances, as previously detailed [85]. Their pairwise relatedness was quantified using the method proposed by Wang [86] and implemented in the R package *related* v. 0.8 [87], as indicated previously [88]. Results were visualized using the R package *pheatmap* v.1.0 [89].

### SNVs selection for high-throughput genotyping

A core set of 56 SNVs representing the complete branching pattern of the inferred phylogenetic network were selected for further tests (Table S2). When possible, these SNVs were chosen from different chromosomes to better represent the overall genomic diversity. IGV v.2.3 [36] was used to manually confirm the genotype of the 50 ‘Tempranillo’ samples at these 56 SNVs, as well as to corroborate the lack of SNVs and/or INDELs nearby the targeted SNVs. Then, a minimum of 60 bp both upstream and downstream of each targeted SNV position were retrieved from the reference ‘Benedicto’ haplophase assembly sequence by means of the getfasta utility of BEDtools version 2.31.0 [90] (Table S2). This information was subsequently provided to the Sequencing and Genotyping Unit of the Universidad del País Vasco (UPV/EHU) to custom-design an intra-varietal genotyping assay for ‘Tempranillo’, using Fluidigm technology. This approach was based on the preamplification of the region flanking the SNV of interest through the specific target amplification (STA) primer, followed by the detection of the different alleles through the combination of the locus-specific primer (LSP) and the allele-specific primers ASP1 and ASP2 (primers are available in the Table S2).

### Wide high-throughput genotyping of ‘Tempranillo’ grapevines

For validation purposes, the intra-varietal Fluidigm genotyping assay designed for ‘Tempranillo’ was firstly tested in the DNAs of the grapevines used for SNV selection. Genotyping was performed using Fluidigm technology, and genotype assignation was done considering only two options per SNV: heterozygous or homozygous for the reference allele. Fluidigm results indicated that all the tested samples showed the genotype called from the Illumina WGR sequencing data at all SNVs, validating this approach (Table S4). Consequently, this approach was used for the high-throughput genotyping of a wide set of ‘Tempranillo’ grapevines (Table S1) using the same Fluidigim technology and procedures.

### Phylogenetic analyses

SNV data was used to infer the phylogenetic network and phylogenetic clustering between genotypes, using the median-joining method implemented in PopART v.1.7 [91], and by the neighbor-joining method available in MEGA X v.11.0 [92], respectively.

### LoH analysis in grey-berried ‘Tempranillo’ somatic variants

The ten grapevines of the grey-berried somatic variant ‘Tempranillo Royo’ included in this work were subjected to a LoH analysis on chromosome 2 as a proxy to identify hemizygous deletions causing the reduced anthocyanin phenotype. To this aim, 48 SNPs located along this chromosome and heterozygous in ‘Tempranillo Tinto’ were screened, as described in Rodríguez-Lorenzo et al. [93] and using Fluidigm technology for high-throughput genotyping. One plant of the original black-berried ‘Tempranillo Tinto’ (Tempranillo-063) was used as reference to confirm the heterozygosity at the 48 SNPs. Similarly, we included one plant of the white-berried somatic variant ‘Tempranillo Blanco’ (Tempranillo-174) with known LoH pattern [8]. LoH screenings were done in DNAs extracted from leaves (formed from L1 and L2 meristem cell layers) and adventitious roots (formed from the L2 meristem cell layer), to detect independent mutations in L1 and L2 cell layers involved in the loss of berry colour in chimeric grey-berried cultivars. Genotyping results from both tissues were compared for detecting LoH regions, and then used to define the chromosome 2-LoH genetic pattern of each grapevine and its possible periclinal chimerism.

## Supplementary Material

Web_Material_uhaf237

## Data Availability

Raw sequencing data generated for this work can be found in the NCBI SRA database as BioProject ID PRJNA1224223.
