# Supplementary material for: Genome resequencing and custom genotyping elucidates the origin and dissemination history of an emblematic grapevine cultivar, ‘Tempranillo Tinto’
Source: Hortic Res. 2025 Sep 3;12(12):uhaf237. doi: 10.1093/hr/uhaf237 (PMC12679915; doi:10.1093/hr/uhaf237)
Supplement: Web_Material_uhaf237 [file web_material_uhaf237.zip › Figure S1_Tello.docx]

**Genome resequencing and custom genotyping elucidates the origin and dissemination history of an emblematic grapevine cultivar, ‘Tempranillo Tinto’**

Javier Tello, Pablo Carbonell-Bejerano, Rafael Torres-Pérez, Yolanda Ferradás, Carolina Royo, Javier Portu, José Félix Cibriáin, Juan Carlos Oliveros, Javier Ibáñez, José Miguel Martínez-Zapater

**
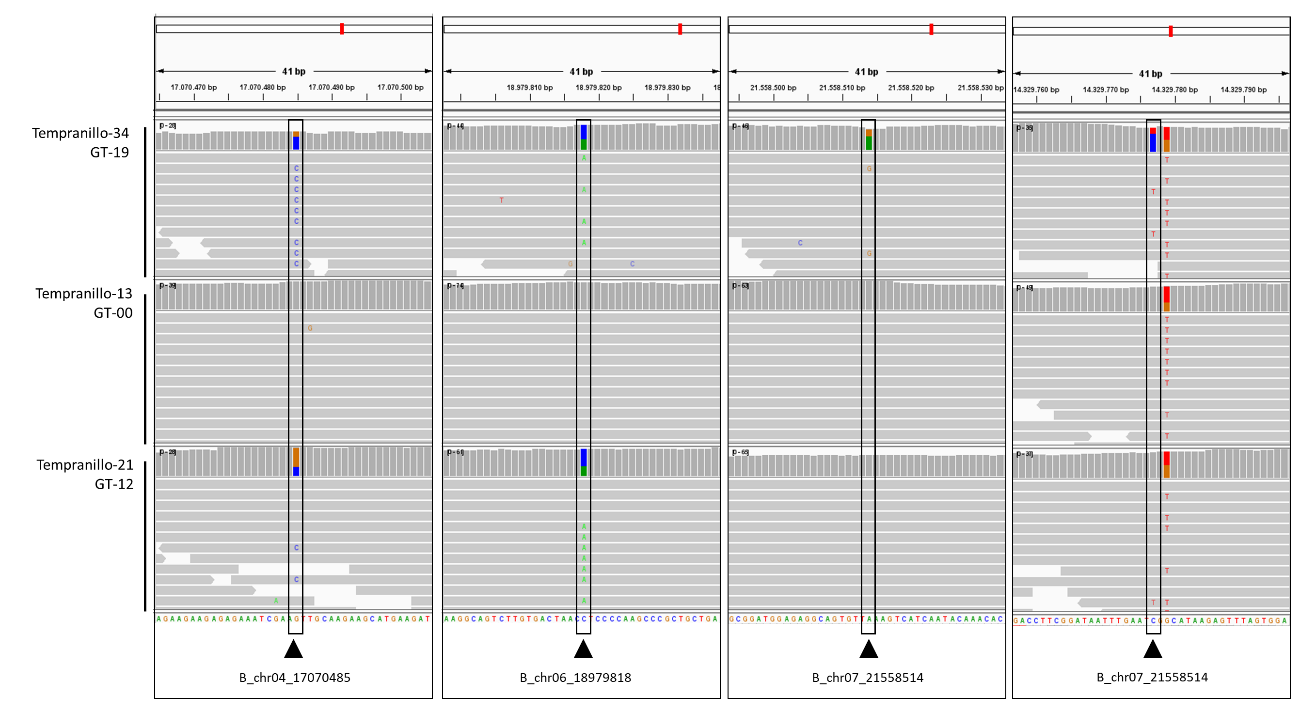
**

**Figure S1**. IGV screenshots of Tempranillo-34 (GT-19), Tempranillo-13 (GT-00), and Tempranillo-21 (GT-12) at the four SNVs (B_chr04_17070485, B_chr06_18979818, B_chr07_21558514, B_chr07_21558514) that determine the three major clades obtained by phylogenetic analyses.
