# Supplementary material for: Genome resequencing and custom genotyping elucidates the origin and dissemination history of an emblematic grapevine cultivar, ‘Tempranillo Tinto’
Source: Hortic Res. 2025 Sep 3;12(12):uhaf237. doi: 10.1093/hr/uhaf237 (PMC12679915; doi:10.1093/hr/uhaf237)
Supplement: Web_Material_uhaf237 [file web_material_uhaf237.zip › Figure S2_Tello.docx]

**Genome resequencing and custom genotyping elucidates the origin and dissemination history of an emblematic grapevine cultivar, ‘Tempranillo Tinto’**

Javier Tello, Pablo Carbonell-Bejerano, Rafael Torres-Pérez, Yolanda Ferradás, Carolina Royo, Javier Portu, José Félix Cibriáin, Juan Carlos Oliveros, Javier Ibáñez, José Miguel Martínez-Zapater

**
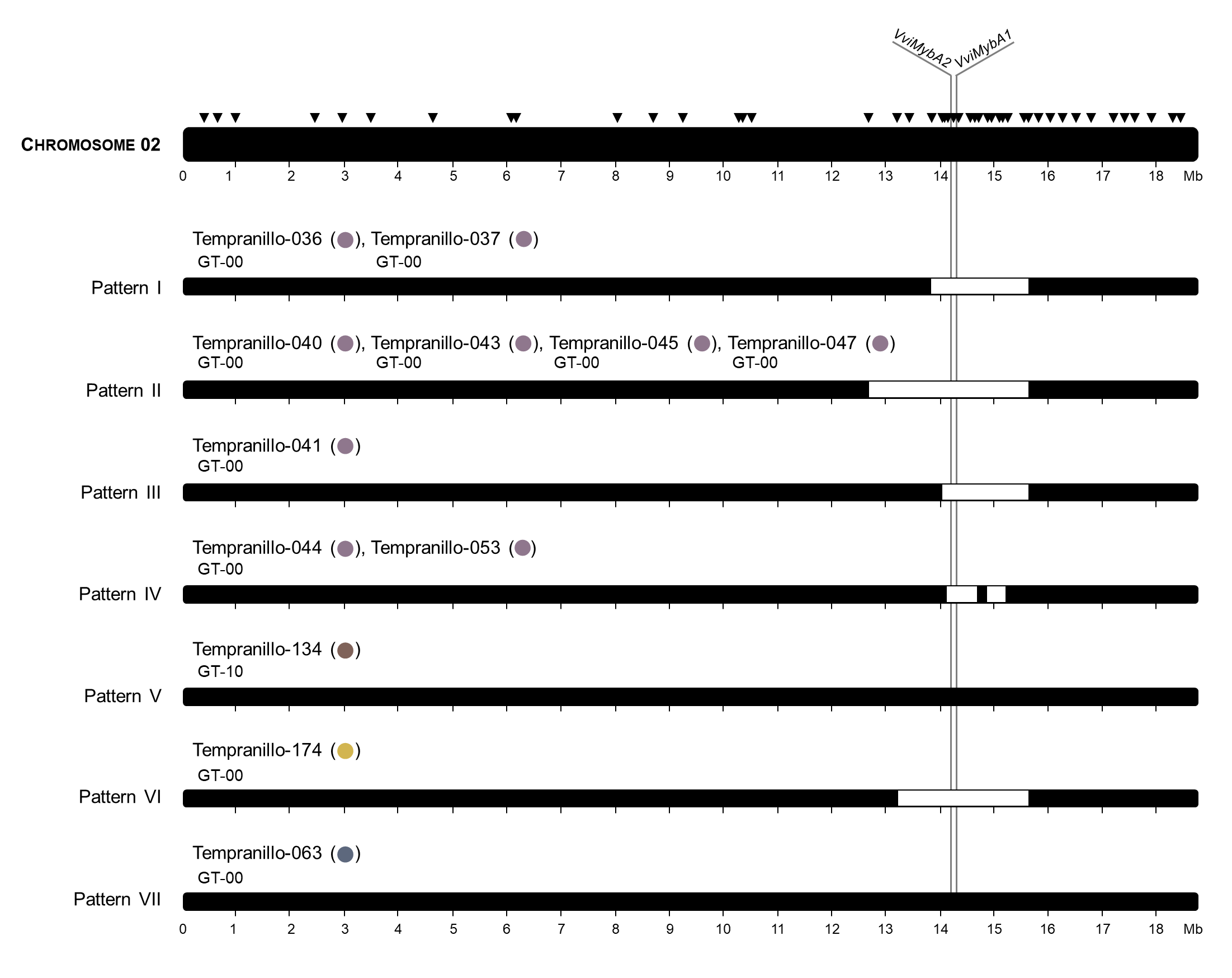
**

**Figure S2**. Genetic patterns of loss of heterozygosity (LoH) found at 48 SNPs of chromosome 2 in Tempranillo somatic variants with reduced anthocyanin pigmentation in the berry skin. The analysis included ten grey-berried grapevines of ‘Tempranillo Royo’, one white-berried grapevine of ‘Tempranillo Blanco’, and one black-berried grapevine of ‘Tempranillo Tinto’. The genomic position of the 48 SNPs screened along chromosome 2 are graphically represented as black triangles. The genomic position of *VviMybA1* and *VviMybA2* (the two main causal genes of the berry color locus of *Vitis vinifera* L.) are also indicated. For each grapevine, its LoH genetic pattern is indicated (I-VII), as well as its berry colour. LoH segments indicating the presence of hemizygous deletion(s) in each genetic pattern are graphically shown as white boxes over a black line, which represents chromosome 2.
