# Supplementary material for: Genome resequencing and custom genotyping elucidates the origin and dissemination history of an emblematic grapevine cultivar, ‘Tempranillo Tinto’
Source: Hortic Res. 2025 Sep 3;12(12):uhaf237. doi: 10.1093/hr/uhaf237 (PMC12679915; doi:10.1093/hr/uhaf237)
Supplement: Web_Material_uhaf237 [file web_material_uhaf237.zip › Figure S3_Tello.docx]

**Genome resequencing and custom genotyping elucidates the origin and dissemination history of an emblematic grapevine cultivar, ‘Tempranillo Tinto’**

Javier Tello, Pablo Carbonell-Bejerano, Rafael Torres-Pérez, Yolanda Ferradás, Carolina Royo, Javier Portu, José Félix Cibriáin, Juan Carlos Oliveros, Javier Ibáñez, José Miguel Martínez-Zapater

**
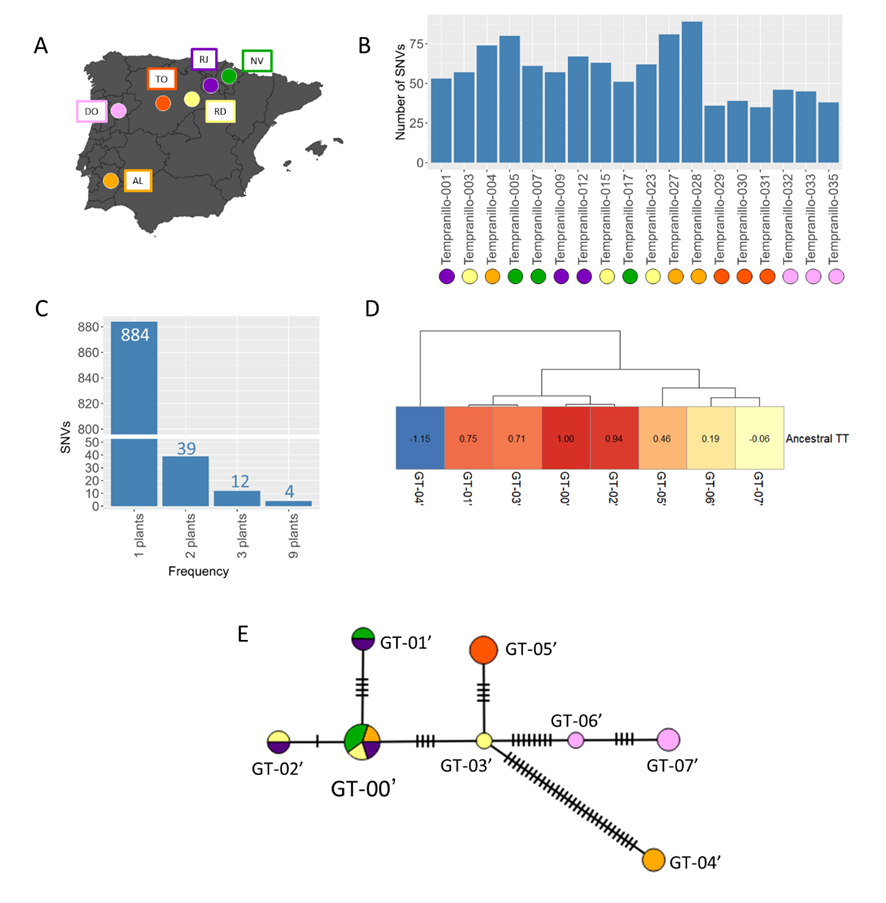
**

**Figure S3**. Phylogenetic analysis of eight ‘Tempranillo’ genotypes (GT-00’ to GT-07’) based on whole-genome resequencing (WGR) data from 18 ‘Tempranillo Tinto’ grapevines collected across six winemaking regions in Spain and Portugal (three grapevines per region). **(A)** Geographic location of the six winemaking regions, coded as described in Table 1. **(B)** Number of single nucleotide variants (SNVs) detected per grapevine based on a variant calling performed on WGR data from 18 ‘Tempranillo Tinto’ grapevines. **(C)** Number of plant-specific and shared SNVs. **(D)** Coefficients of relatedness between each genotype and an inferred “Ancestral Tempranillo” (Ancestral TT) genotype inferred from ‘Benedicto’ and ‘Albillo Mayor’ genomic data. **(E)** Phylogenetic relationships among the eight ‘Tempranillo’ genotypes.
